# RSV immunisation in infants: weighing the options

**DOI:** 10.1016/j.lanepe.2024.100867

**Published:** 2024-02-08

**Authors:** Felix Günther, Frank G. Sandmann

**Affiliations:** Immunisation Unit – Team Vaccine/VPD Modelling, Department for Infectious Disease Epidemiology, Robert Koch Institute, Berlin, Germany

The human Respiratory Syncytial Virus (RSV) is a common cause of lower respiratory tract infections worldwide, which can cause severe bronchiolitis with fatal outcome. About 1 in 55 healthy-born children in Europe are hospitalised because of RSV in the first year of their life,^1^ and an estimated 1 in 30 deaths in children aged 1–6 months were attributable to RSV globally.^2^ To protect the infants, two new medical products were licensed recently: (a) single-dose long-acting monoclonal antibodies, and (b) maternal vaccines protecting infants through the transplacental transfer of antibodies. National Immunisation Technology Advisory Groups (NITAGs) worldwide currently consider recommending universal infant RSV immunisation using such products.

In this issue of the *Lancet Regional Health—Europe*, Hodgson et al. examined the (cost-)effectiveness of administering long-acting monoclonal antibodies to healthy-born infants versus vaccinating pregnant women between 24 and 36 weeks gestational age in the UK – both supplementing the current practice of multi-dose short-acting monoclonal antibodies in high-risk infants.^3^ The results indicate that the interventions could substantially reduce the RSV burden in infants aged 0–2 months by 30%–76%, with a comparable population-level impact per dose. Seasonal programmes appeared slightly more efficient than a year-round programme. Based on cost-effectiveness considerations and a value of £20,000 per quality-adjusted life year gained, long-acting monoclonal antibodies were always preferable up to a price and delivery cost of £35 per dose, while up to £85 it depended on the costs of maternal vaccination. If both new interventions cost each above about £90, retaining the current practice was the most cost-effective of the analysed scenarios.

In their analysis, Hodgson et al. combined statistical and mathematical modelling with multiple data sources in different analysis steps, including novel Bayesian modelling of the level and duration of protection from efficacy trial data; 10-year forward simulations of future RSV dynamics under the different immunisation scenarios with their previously-published dynamic-transmission model that was calibrated to RSV-confirmed cases from sentinel laboratory surveillance in 2010–2017 using Bayesian methods; and subsequent health-economic evaluation using published estimates for England and Wales. Based on this study design, the authors addressed common challenges in modelling studies like (i) specifying an adequate model representing key processes in transmission and epidemiology, (ii) estimating key parameters governing transmission dynamics like the force of infection and the protection from immunisation, and (iii) specifying realistic assumptions on frequencies of disease endpoints and associated costs. Thereby, the authors improve on previous RSV modelling with similar results that ignored indirect effects or used different waning assumptions.^4^^,^^5^

The analysis also informed the NITAG in the UK, the Joint Committee on Vaccination and Immunisation (JCVI), illustrating its public health relevance. JCVI recommended either new product for infant immunisation without preference, but with preference for a year-round programme given operational reasons and to ensure high uptake.^6^ Outside of the UK, analysts in other countries with different RSV dynamics and healthcare systems may benefit from the transparent reporting and published source code.^3^

Further questions remain on aspects that were outside the scope of this work. For example, the high uptake observed for the long-acting monoclonal antibody above 90% in one region of Spain in end-2023^7^ raises questions for the wide-scale availability of the product if recommended in more countries at an equally-high acceptance. Related questions remain for global equity and supply, particularly in low- and middle-income countries bearing the largest RSV burden.^2^ Moreover, the possibly increased risk of premature births for the maternal RSV vaccination requires further research and must be carefully weighed up (e.g., in the USA maternal vaccination was recommended only during 32–36 weeks of gestational age^8^). Vaccines have been licensed for older adults recently too, but the duration of the protection remains uncertain and hence the frequency of elderly vaccination programmes and the most cost-effective age of eligibility (e.g., in the USA a single dose using shared clinical decision-making was recommended for ages 60 years and older,^8^ and JCVI recommended a single dose for ages 75 years and older^6^). Similarly, paediatric vaccines can be expected to become an important strategy once they are licensed, particularly given the potential age shift of the RSV burden from infants into children aged 1 year with universal infant immunisation.^3^ More RSV vaccine candidates seek licensure, and they may be combined with other vaccines.^9^ Further questions remain regarding the (future) interaction of RSV and other pathogens; although at least the usual endemic RSV patterns have returned in many high-income countries after the disruptions from the coronavirus disease 2019 (COVID-19) pandemic.^10^

In conclusion, it is prime time for weighing of the options of RSV immunisation. Equally important will be setting up accompanying epidemiological studies to analyse the effects of any implemented strategies as RSV prevention enters the limelight of public health this decade.

## Contributors

FG: conceptualisation, writing—original draft, and writing—review & editing. FGS: conceptualisation, supervision, and writing—review & editing.

## Declaration of interests

The Robert Koch Institute (RKI) received funding for a modelling project of RSV immunisation strategies in Germany from the Federal Joint Committee (grant number: 01VSF18015). FGS holds an honorary, non-remunerated role at the London School of Hygiene and Tropical Medicine (LSHTM). The views expressed in this Comment are exclusively those of the authors.
